# Why is the prevailing model of joint manipulation (still) incorrect?

**DOI:** 10.1186/s12998-022-00460-2

**Published:** 2022-12-09

**Authors:** David W. Evans

**Affiliations:** 1 Centre of Precision Rehabilitation for Spinal Pain, School of Sport, Exercise and Rehabilitation Sciences, University of Birmingham, Edgbaston, Birmingham, B15 2TT UK; 2 Research Centre, University College of Osteopathy, London, UK

**Keywords:** Manual therapy, Manipulation, Joint, Metacarpophalangeal, Cavitation, Definition, Model

## Abstract

For manipulation, this paper addresses arguably the most fundamental question that can be asked about any therapeutic intervention: *what is it*? In answering this question, this paper presents the prevailing model of joint manipulation (of Sandoz) and explains why this influential model is fundamentally flawed. The early research on ‘joint cracking’ that led to the development of this model is described in chronological order, alongside how this research was misinterpreted, which gave rise to the model’s flaw. Of concern, the flaw in this model makes worrying predictions that could lead to dangerous clinical decisions. Understandably, these predictions have attracted criticism over the use of manipulation as a therapeutic intervention. A corrected model, first published by Evans and Breen more than 15 years ago, is then presented and explained. Unlike the flawed model, this corrected model makes predictions in line with all available empirical data and additionally provides reassuring answers to critics. Many current definitions of manipulation have inherited the flaw from Sandoz’s model. Hence, a better, empirically derived definition, consistent with the corrected model, is now required.

## Introduction

If one attempts to read through the extensive literature that relates to manipulation (in the manual therapy context), it becomes very noticeable that literally dozens of definitions and descriptions have been proposed [1]. Often, these definitions conflict with one another and on occasion can be found in unexpected places [2], such as within primary legislation [3, 4]. Representative examples of such definitions are presented in Table 1.

**Table 1:** Examples of existing definitions of manipulation

Source	Details	Definition
Sandoz [5]	Expert opinion, Switzerland	“A passive, manual manoeuvre during which an articular element is suddenly carried beyond the usual, physiological limit of movement without however exceeding the boundaries of anatomical integrity. The usual but not obligate characteristic of an adjustment is the thrust which is a brief, sudden and carefully dosed impulsion delivered at the end of the normal passive range of movement and which is usually accompanied by a cracking noise.”
Nyberg [6]	Expert opinion, USA	“Thrust manipulation is the use of high velocity, low amplitude motion delivered at the end of the restricted physiologic limit of a joint’s range of motion.”
Gatterman and Hansen [7]	Consensus of chiropractors, international	“A manual procedure that involves a directed thrust to move a joint past the physiological range of motion, without exceeding the anatomical limit”
International Federation of Orthopaedic Manipulative Therapy [8]	Professional organisation, international	“A passive, high velocity, low amplitude thrust applied to a joint complex within its anatomical limit* with the intent to restore optimal motion, function, and/or to reduce pain *anatomical limit: Active and passive motion occurs within the range of motion of the joint complex and not beyond the joint’s anatomic limit.”
Government of Ontario [3]	Primary legislation, Canada	“Moving the joints of the spine beyond a person’s usual physiological range of motion using a fast low-amplitude thrust.”

The composition of definitions listed in Table 1 is worthy of attention. Firstly, most of them commit to specifying *a joint* as the unit of manipulation, which deserves credit [1]. Beyond this, however, one can easily find flaws. The term *thrust*, for example, is used in the colloquial sense and is therefore inappropriate for a formal definition. Thrust is a reaction force (i.e., a force that acts in the opposite direction to the line of action of an applied force) described quantitatively by Newton's third law of motion, which states that all forces between two objects exist in equal magnitude and opposite direction. Thrust is produced by a rocket’s engine when it rapidly expels the mass of its burned fuel in one direction, which simultaneously creates a reaction force that propels the rocket in the opposite direction. If the term must be used in the context of manipulation, thrust is technically the reaction force from the recipient to the practitioner, not the other way around.

Several definitions include a clause stating that movements induced during a manipulation will stop short of causing tissue damage. Such clauses have been phrased as “*without exceeding the boundaries of anatomical integrity*” [5], *“without exceeding the anatomical limit*” [7], or “*within its anatomical limit*” [8]. However, what if this anatomical limit *is* breached and tissues *are* damaged? Is this no longer a manipulation? This seems just too convenient and makes these definitions appear contrived. As would be the case with any other intervention (e.g., surgery), a manipulation that induces tissue damage or any other form of harm must still be a manipulation, irrespective of the (unintended) adverse outcomes.

Some unusual terms have been used within definitions of manipulation, as can be seen in Table 1. The term arguably requiring most explanation is *physiological range of motion*, which suggests that there is at least one other *non-physiological* range of motion. To understand the origins and full meaning of this, and the other unusual terms within these definitions, we need to look closely at two important studies, which first requires a detour to post-war London.

## Cracking joints

**Fig. 1:**
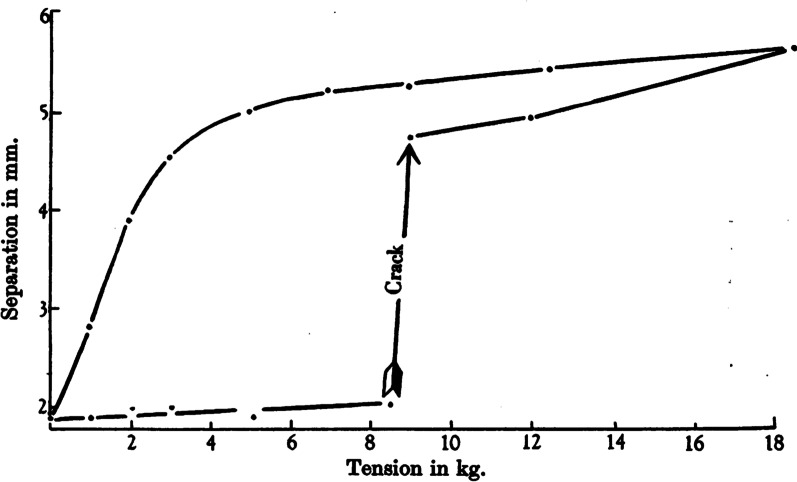
Separation of the articular surfaces of an MCP joint in response to axial loading. The original caption of this figure, reproduced from Roston and Wheeler Haines [9], was “Record of the separation of the bones in a typical cracking joint”

The first bioengineering study that looked at the relationship between joint movement and the phenomena of joint ‘cracking’ was published in 1947, by two physicians at the renowned St. Thomas’s Hospital Medical School in London. In their landmark study [9], J.B. Roston and R. Wheeler Haines, understudies of the famous musculoskeletal physician James Cyriax, simultaneously measured three important things: the magnitude of a gradually increasing ‘traction’ force applied, via a pulley system on which weights were incrementally applied, to ‘pull’ a finger along its long axis (i.e., the force was applied perpendicular to the joint surfaces). The consequential separation (gapping) between the two articular surfaces of a metacarpophalangeal (MCP) joint was measured using x-ray radiography from directly above the hand (i.e., looking through the joint space), and the moment was noted when a ‘cracking’ sound was produced. For the first time, they published the now classic diagram displayed in Fig. 1.

To orientate ourselves with this important diagram, we can first observe that neither of the lines intersecting the vertical axis do so at zero. This is because the separation between the joint surfaces, represented by the vertical axis, was taken from a series of x-ray images (radiographs) that do not show articular cartilage; this particular MCP joint must have possessed approximately 1 mm of cartilage on each articular surface, causing the starting point to be just under 2 mm. The initiation of joint surface separation is described by the relatively horizontal, straight line that extends just above the horizontal axis until approximately 8 kg mass (80 N of force) is applied to the pulley system; the relatively flat slope (gradient) of this line shows that little joint surface separation is occurring as the applied force increases. Whatever is providing resistance until 8 kg is therefore fairly stiff. Suddenly, this line changes from being near horizontal to being near vertical, where Roston and Wheeler Haines explicitly indicate that this abrupt change is accompanied by an audible ‘crack’. After this crack, with increasing load the line returns to a near horizontal gradient once again, until it reaches a maximum separation of approximately 18 mm; a relatively large separation for an MCP joint! Collectively, the three phases of this ‘outward’ path describing increasing joint surface separation form something of a ‘Z’ shape. This Z-shaped path is extremely important, as we shall see later on. Attentive readers will also notice that a second path is drawn on the diagram reproduced in Fig. 1; this represents the separation between the joint surfaces shrinking as applied force is reduced back to zero. This ‘return’ path is very different; it is curved and does not overlap the original outward path.

**Fig. 2:**
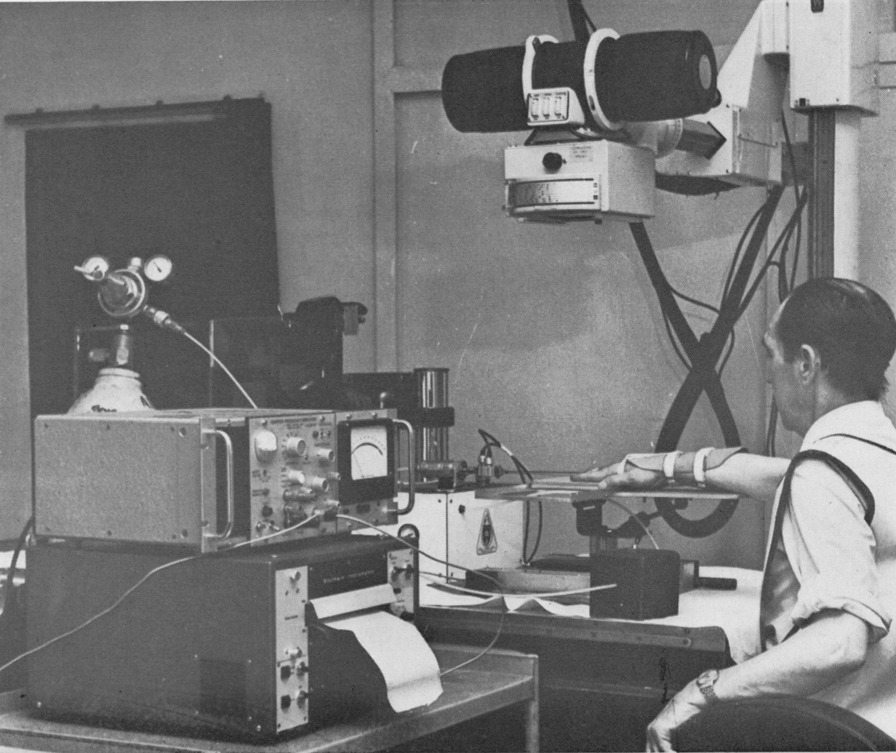
Experimental setup used to simultaneously measure MCP joint surface separation and applied force. The original caption of this figure, reproduced from Unsworth et al. [10], was “A machine designed to ‘crack’ the metacarpophalangeal joints of human subjects”

**Fig. 3:**
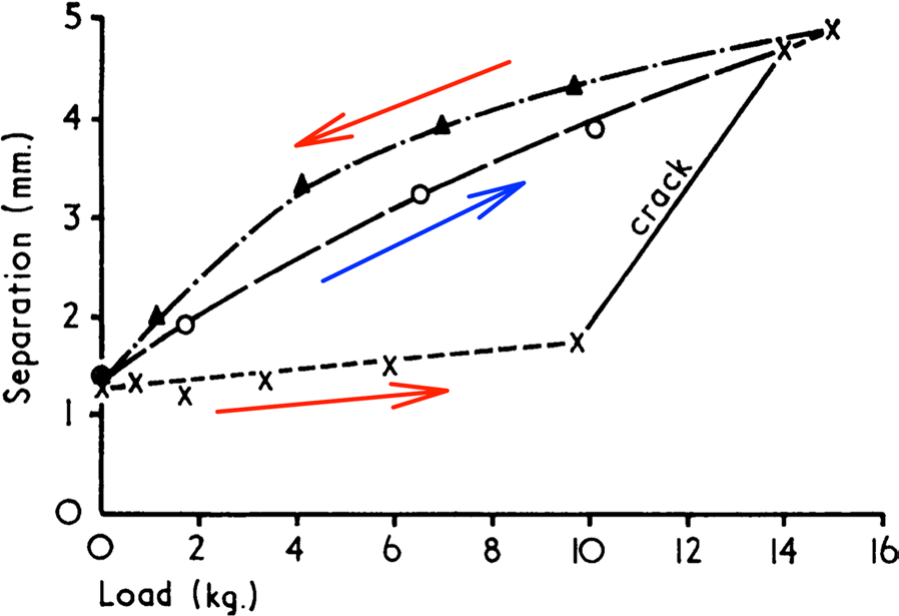
Load–displacement curves of MCP joint surface separation. The original caption of this figure, reproduced with additional annotations from Unsworth et al. [10], was “Typical load–separation curve for a cracking joint”

Roston and Wheeler Haines did not just provide the first example of the above diagram; they discussed the likely mechanism of the cracking phenomenon, and rightly implicated the intra-articular synovial fluid as crucial to this. However, it wasn’t until the late 1960s where a group of engineers from the University of Leeds, again in England, brought their considerable expertise and knowledge of human joint tribology to the phenomenon of joint cracking and produced the undisputed authoritative study on the subject [10]. The methodology employed by the Leeds group (Fig. 2) was very similar to that of the London study [9], and the key figure published within their results (Fig. 3) was strikingly similar to that presented in Fig. 1. The archetypal Z-shaped outward path associated with joint cracking is again present, as is the smooth return path (marked by red arrows in Fig. 3). However, there was one ingenious addition in the Leeds study: after the applied force had returned to zero, the force was once again increased for another loading cycle (indeed the authors report preforming multiple loading cycles). It can be seen that the second outward path (marked by the blue arrow in Fig. 3) is smooth like the return path, although these don’t quite overlap. This addition demonstrated that the Z-shaped path is a once-only event, at least for an undefined time period of “about 20 min” [10] following its first occurrence.

## Cavitation

**Fig. 4:**
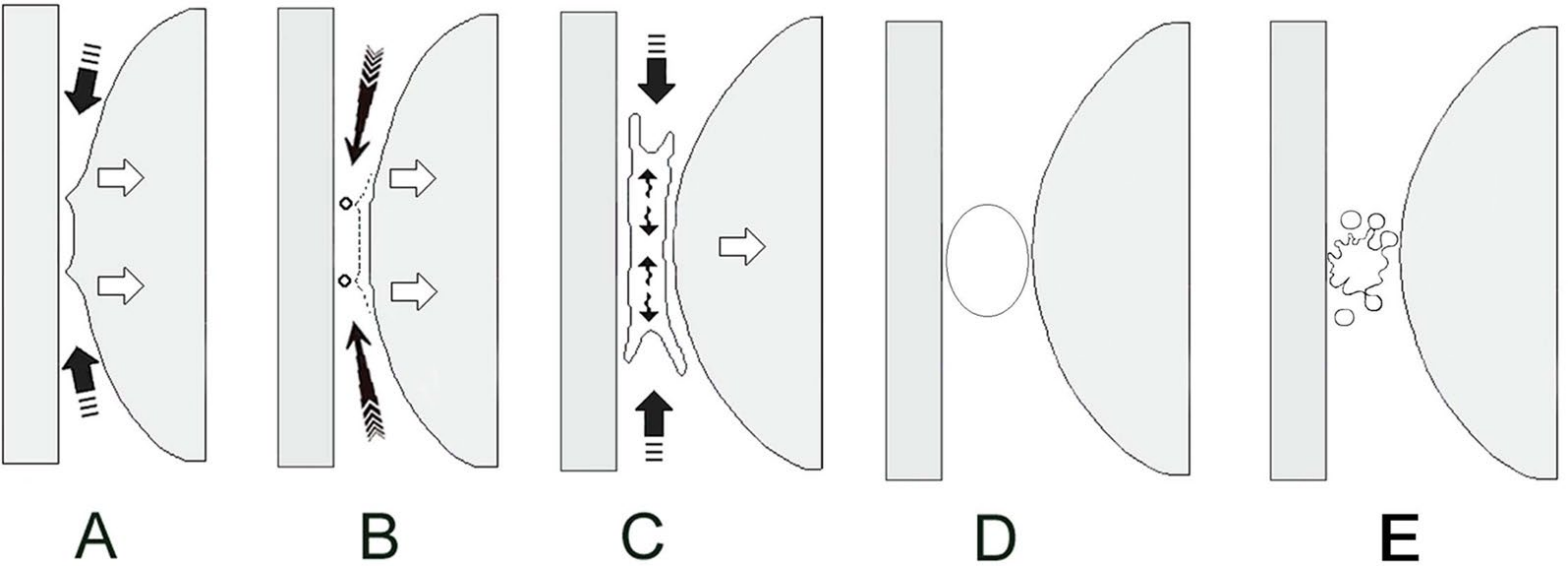
Cavitation occurring between the articular surfaces of synovial joints. Based on Chen et al. [12]

The Leeds group also made a monumental step with regards to explaining the mechanism underlying the previously mysterious cracking event and the associated Z-shaped force–time path; for the first time, *cavitation* within synovial fluid was explicitly named as being responsible for the audible cracking phenomenon. Cavitation is the formation and activity of bubbles in fluid through the local reduction of pressure within fluid. During joint cracking, this pressure reduction is caused by the separation of the joint surfaces [10], which increases the volume within the closed joint cavity (Boyle’s law). The fluid pressure is reduced to a negative value, producing tension [11]. The reduced pressure must reach a critical threshold, after which the fluid will fracture [12] to form a bubble from gases already dissolved in the synovial fluid [10, 13]; these gases are believed to consist mostly of carbon dioxide, although this has only ever been measured indirectly [10]. The nascent bubble grows very rapidly to a maximum size, before immediately and violently collapsing as the synovial fluid rushes into this lower pressure region [12] (Fig. 4). These high-energy events are responsible for the characteristic cracking noise. This mechanism also provided an explanation for the *refractory period* first described by Roston and Wheeler Haines [9]; following the initial bubble collapse, it takes time for the liberated gases to fully dissolve back into the synovial fluid. This gas is likely to remain in solution as a cloud of more stable micro-bubbles [13]. New bubbles cannot be formed until this gas re-dissolves back into the synovial fluid; attempts to do so by distracting the joint simply expand these existing micro-bubbles and produce no cracking noise.

## The Sandoz model of manipulation

**Fig. 5:**
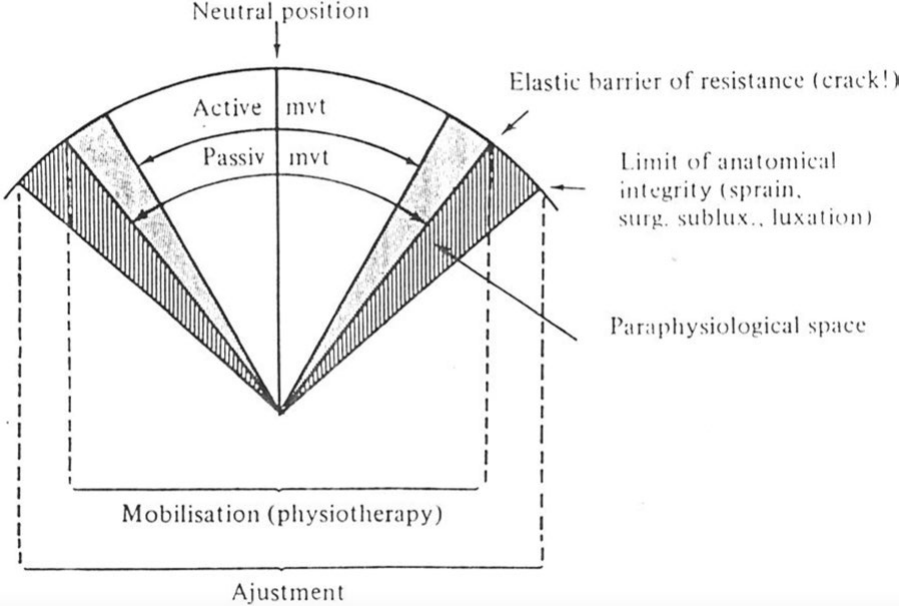
Sandoz’s original model of joint manipulation. The original caption of this figure, reproduced from Sandoz [5], was “Joint mobilisation & adjustment”

The seminal work of Roston and Wheeler Haines in London and then of Unsworth, Dowson and Wright in Leeds explained *all* important aspects of joint cracking and gave significant clues for the likely therapeutic mechanisms of action of manipulation. Unfortunately, few clinicians appear to have gained their knowledge first-hand from these two ground-breaking studies; instead, it seems that most did so second-hand through the interpretation of Raymond Sandoz, a French-Swiss chiropractor who published a handful of influential papers on manipulation [5, 14, 15]. By far the most enduring legacy of Sandoz’s published work was his *model of joint manipulation* (Fig. 5), the ideas for which he explicitly attributed to the results of the London and Leeds studies.

As can be seen in Fig. 5, Sandoz attempted to define different interventions and their effects by ranges and limits of *motion*; in this sense, he followed a similar approach to well-known Australian physiotherapist, Geoffrey Maitland [16]. However, Sandoz’s model is important primarily because it has become conventional wisdom. Indeed, the wording of most current definitions, including those listed in Table 1, is derived from Sandoz’s two-dimensional arc-shaped model, his terminology and conclusions [1, 2]. There is, however, one problem with this: Sandoz got the most important element of his model totally and utterly wrong!

For those that learned Sandoz’s model during their professional training or have some investment in the ideas underlying his model, please don’t despair. Canny observers (e.g., Singh and Ernst [17]) have noticed that Sandoz’s model makes some worrying predictions (Table 2). Unfortunately, these observers are *not* mistaken. Most concerning of the model’s predictions is that the peak manipulation force is intended to move the joint beyond *any* resistance met at the end of its passive range of rotation. Doing so would, of course, be very dangerous. Sandoz undoubtedly knew of such risks which he incorporated into his *limit of anatomical integrity*, referring to joint capsule and ligamentous injury (“sprain”). Without doubt, this is the reason he felt the need to (conveniently) place his *elastic barrier of resistance*—sometimes referred to as the *physiological barrier*—in the way of such injury. Few have publicly questioned Sandoz’s model; instead, attempts have been made to patch it up [2], or explain away its predictions in terms of high-threshold afferent stimulation [15, 18–23], but these attempts have been unsuccessful in removing its end-range danger.

**Table 2:** Predictions made by Sandoz’s model of joint manipulation compared to the corrected model

Prediction during ‘thrust phase’ of manipulation	Sandoz model (Sandoz 1976) [5]	Corrected model (Evans and Breen 2006) [24]
Joint configuration	Joint is in end-range configuration	Joint is in (or close to) neutral configuration
Line of action of force applied	Parallel to articular surface	Perpendicular to articular surface
Joint motion	Articular surfaces will slide	Articular surfaces will separate (gap)
Articular surface separation (gapping)	Not incorporated in model at all	Maximal availability around neutral configuration, minimal at end-range configuration
Anatomical restraining (capsular-ligamentous) tissues	On maximal tension	On minimal tension
Source of first resistance	Joint capsule and ligamentous tissues	Synovial fluid
Occurrence of cavitation	Beyond end range of physiological range of motion	Within physiological range of motion

## Sandoz’s biggest blunder

**Fig. 6:**
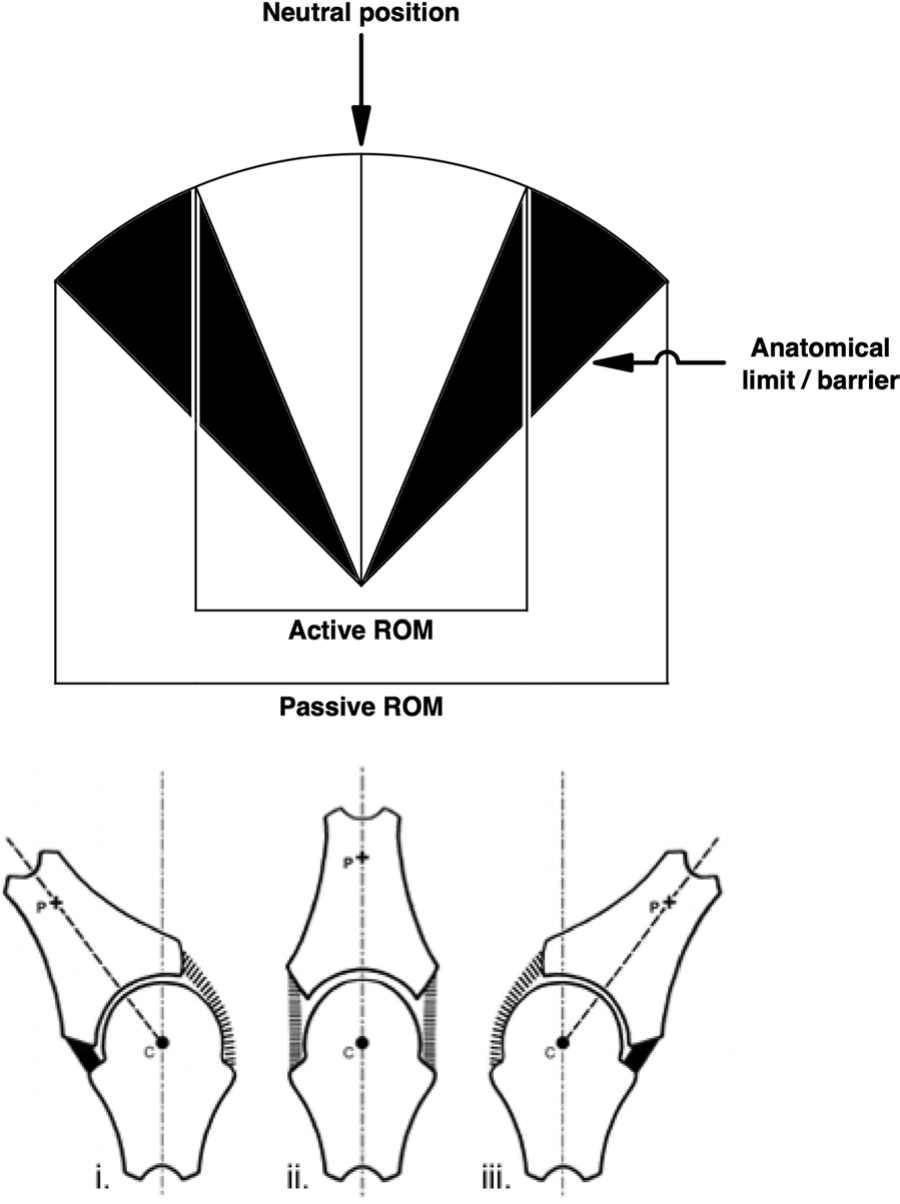
Simplified two-dimensional model of joint motion

Let’s take a closer look at Sandoz’s error and how it arose. Figure 6 shows a simplified version of his symmetrical two-dimensional arcuate model, demarcating active and passive ranges of motion in a single plane. Partitioned synovial joint diagrams are placed beneath to represent the joint configuration at different ranges of motion. At this stage, there is nothing factually incorrect or controversial about this dissected version of Sandoz’s model. It is entirely true that passive stretches will produce larger ranges of motion than can be achieved by active movements along the same plane. This is easily demonstrated with any finger in any direction. In doing so, the increasing range of passive motion will meet growing resistance from anatomical restraints until no further motion is possible, unless tissues fail: this is Sandoz’s *anatomical limit*.

Sandoz’s blunder occurred when he added his *elastic barrier of resistance* to the model. This was the term he used to represent the stiff resistance—known since 1947 to be caused by synovial fluid—that was seen during the first phase of the Z-shaped path (Figs. 1 and 3). Recall that this *elastic barrier* was overcome with a distinctive ‘crack’ (the second phase of the Z), beyond which Sandoz referred to a *paraphysiological space*; the newly available addition to the joint’s range of motion (the third phase of the Z). Observant readers might already have spotted Sandoz’s mistake when looking at Fig. 6 while also recalling how data were collected during both London and Leeds studies (depicted in Fig. 2). For those readers in need of a little more convincing, one important fact must be remembered: every single study of joint cracking in MCP joints, before Sandoz and since, has invoked cavitation by separating articular surfaces through joint distraction—pulling the finger along its long axis—and not through a rotational motion such as that depicted in Sandoz’s model.

From knuckle-crackers, at this point there is typically a comment along the lines of, “hang on, I use joint rotation to crack my knuckles!” This may be true, but your habit will not have been satisfied by a *pure* rotation about a stationary axis; it will only have been fulfilled by rotation *plus* some distraction. To test out the effect of a pure rotational motion, try fully flexing, extending or laterally bending an MCP joint by applying a force at the very the tip of one of your fingers, and you will only *feel* the silent resistance of joint capsules, ligaments and tendons.

With Sandoz’s error now hopefully obvious, if we allow ourselves to retain his terminology the important question to now ask is, ‘where should he have placed his *elastic barrier*?’ Undoubtedly, the resistance from synovial fluid that produces the first (near-horizontal) phase of the Z-shaped path is very real and measurable, but where (and how) should one incorporate it onto the two-dimensional arc-shaped diagram of joint motion that was depicted in Fig. 6?

**Fig. 7:**
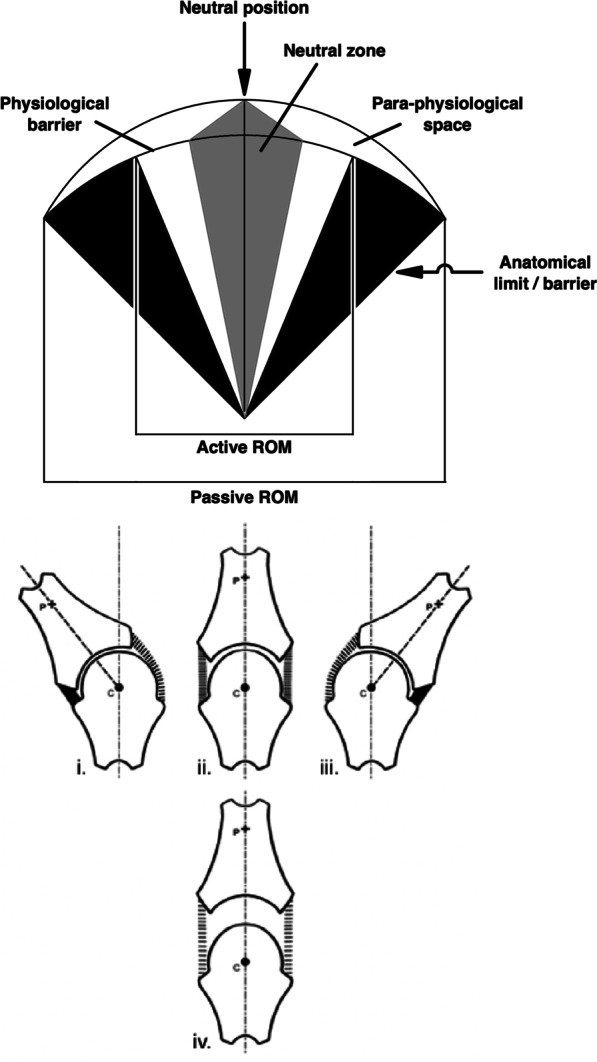
The corrected model of joint manipulation. Based on Evans and Breen [24]

The answer was published in 2006 [24]. Figure 7 reveals the correct location for the *elastic barrier* and the *para-physiological space* beyond. As guided by the partitioned synovial joint diagrams below the corrected arc-shaped model, the relationship between the centre of rotation (*c*) and the arbitrary fixed point (*p*) dictate both the correct location and extent of the *para-physiological space*. It now resides on top of the model, upon the upper border of the arc drawn by the rotational joint motion. While the joint surfaces are in contact, the *para-physiological space* has no area in this two-dimensional diagram (nor volume in a real three-dimensional joint). The space is therefore a *potential* space, akin to that of the pleura, only becoming real and apparent when the surfaces separate. The relative invisibility of this space is most likely why Sandoz missed its true location. Indeed, creating joint surface separation requires a force with a line of action perpendicular to the articular surfaces [1]; in plain sight in Fig. 2, yet must have been overlooked by Sandoz.

Apart from the fact that the corrected model (Fig. 7) now demonstrates joint surface separation (gapping), which in addition to MCP joints has been confirmed to occur in zygapophysial joints during manipulation of the spine [25–27], its most important success is that it predicts that cavitation will occur *within* the joint’s *physiological range of motion* (Table 2). When the joint is orientated in its neutral position, the capacity for articular surface separation *without tissue damage* is maximal, because the joint capsule and surrounding ligaments are in their most lax configuration. However, as the joint rotates around the point (c), moving further from its neutral position and towards its end range of rotation, this capacity diminishes towards zero. Therefore, articular surface separation (and consequently synovial fluid cavitation) will be most efficiently attained when the joint is closest to its neutral configuration [24]. If clinicians prefer to think in terms of *grades* of motion in individual joints [16], the term ‘Grade 0’ (representing proximity to the joint’s neutral position) should arguably be used instead of ‘Grade 5’.

## After Sandoz

**Fig. 8:**
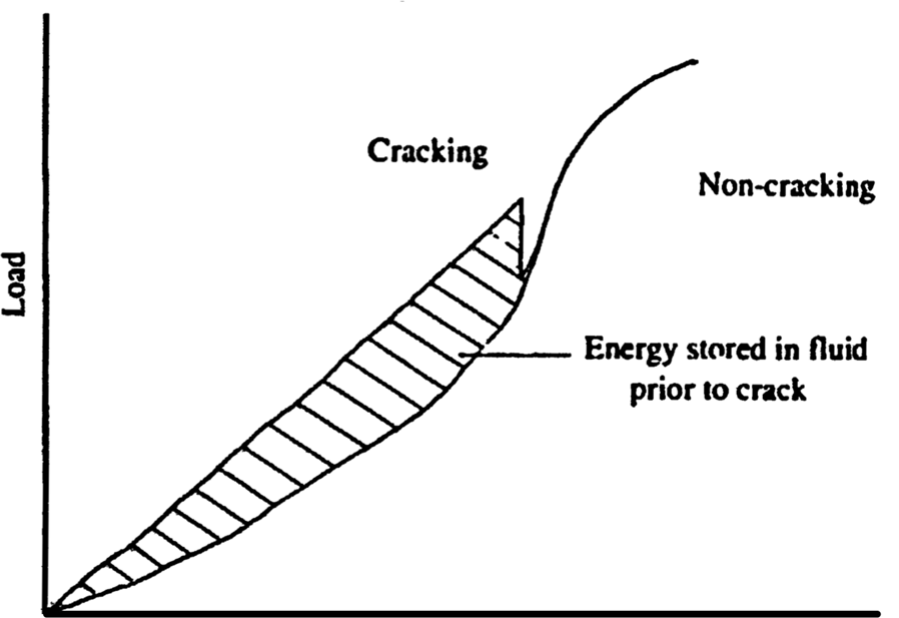
Energy stored within the synovial fluid of an MCP joint during joint distraction Reproduced from Watson et al. [28]

Further details should be added from the evidence for cavitation within synovial joints, which continued to grow unhindered despite the publication of Sandoz’s erroneous model. In the late 1980s, a team of scientists [28] in another city within the British Isles, Belfast in Northern Ireland, built upon the seminal work of the London and Leeds groups, utilising advances in technology available at the time. Their work produced several useful additions to the existing knowledge. Firstly, they inverted the causal process employed by the previous groups, instead using a constant rate of extension (joint surface separation) as the independent variable whilst continually measuring the resistance (load) provided by the synovial fluid as the dependent variable; this focus on the *elastic barrier* provided deeper insights to the Z-shaped path recorded in the previous studies. The diagram that they created (Fig. 8) shows that the linear relationship between load and separation is once again retained until the cracking event, which creates another Z-shaped perturbation. Their other important result was to equate the area between the non-overlapping outward and return paths with the energy stored within the synovial fluid prior to the crack (force multiplied by distance equates to work done by energy). This stored energy is equivalent to the hysteresis curves demonstrated by the previous studies.

**Fig. 9:**
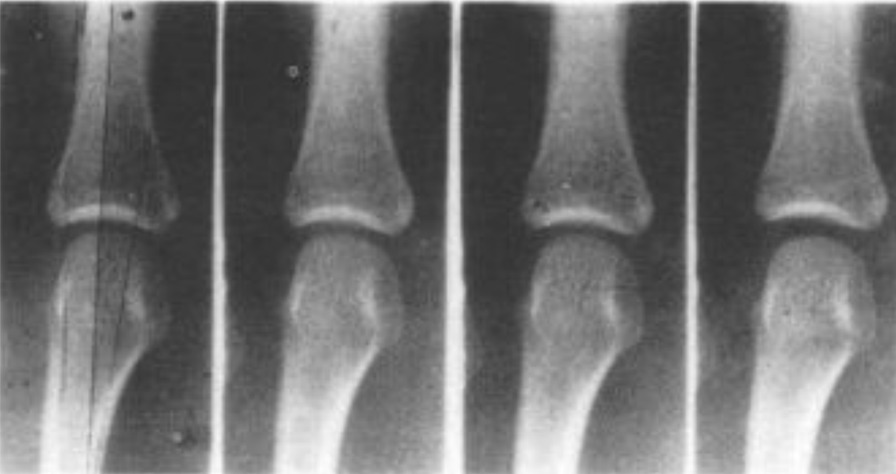
The formation of a bubble during MCP joint distraction. The original caption of this figure, reproduced from Watson and Mollan [29], was “Three frames before and one frame after the MCP joint crack. The frames are separated by approximately 8.3 ms and the bubble has appeared in the joint space between the last two frames”

In addition to their work on energy storage and synovial fluid cavitation, Watson and colleagues [29] also provided the first real-time images of the appearance of bubbles in synovial fluid. Using the technique of cineradiography (high-frequency x-ray), capturing 120 frames per second, they were able to show that a gas bubble had fully formed within the synovial fluid between two of their frames; in other words, within 8.3 ms (Fig. 9).

Despite compelling evidence that cavitation is entirely responsible for joint cracking being available since the 1980s, some clinical commentators have argued a position against this for several decades [30, 31]. Thankfully, more recent studies on synovial joint cracking have caught up with advances in imaging technology, and today provide such unequivocal evidence that even the most ardent cavitation-denialists should by now have changed their minds.

**Fig. 10:**
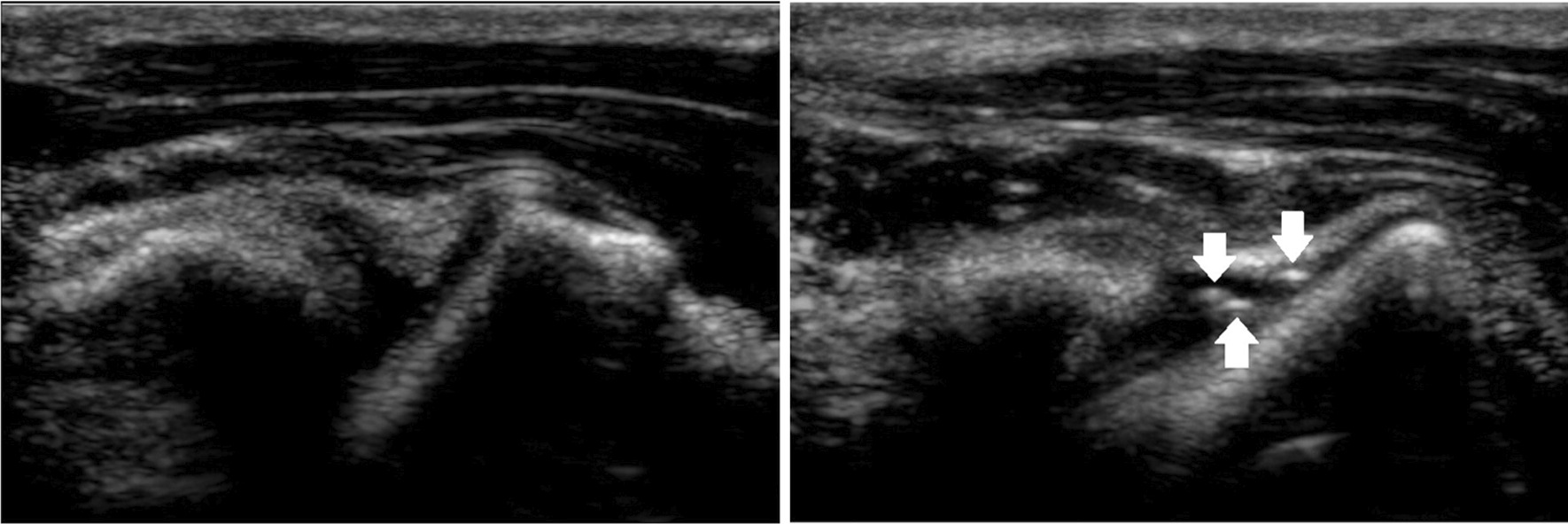
Ultrasound images before and after cavitation in a trapeziometacarpal joint Reproduced from Jones et al. [32]

In 2014, Jones and colleagues from South Africa [32] published the first images of synovial fluid bubbles resulting from cavitation in trapeziometacarpal joints; the thumb’s equivalent of the MCP joint (Fig. 10). Not only did this study provide further evidence for the existence of such bubbles following joint cracking, but it also showed that the refractory period, during which the liberated bubble gases would dissolve back into synovial fluid, could last much more than “about 20 min”, which had been the assumption since the Leeds study [10]. Unfortunately, Jones et al. [32] did not report on the timing of bubble formation, despite acquiring 15,000 sonograph images per second (the highest frame rate recorded to date).

**Fig. 11:**
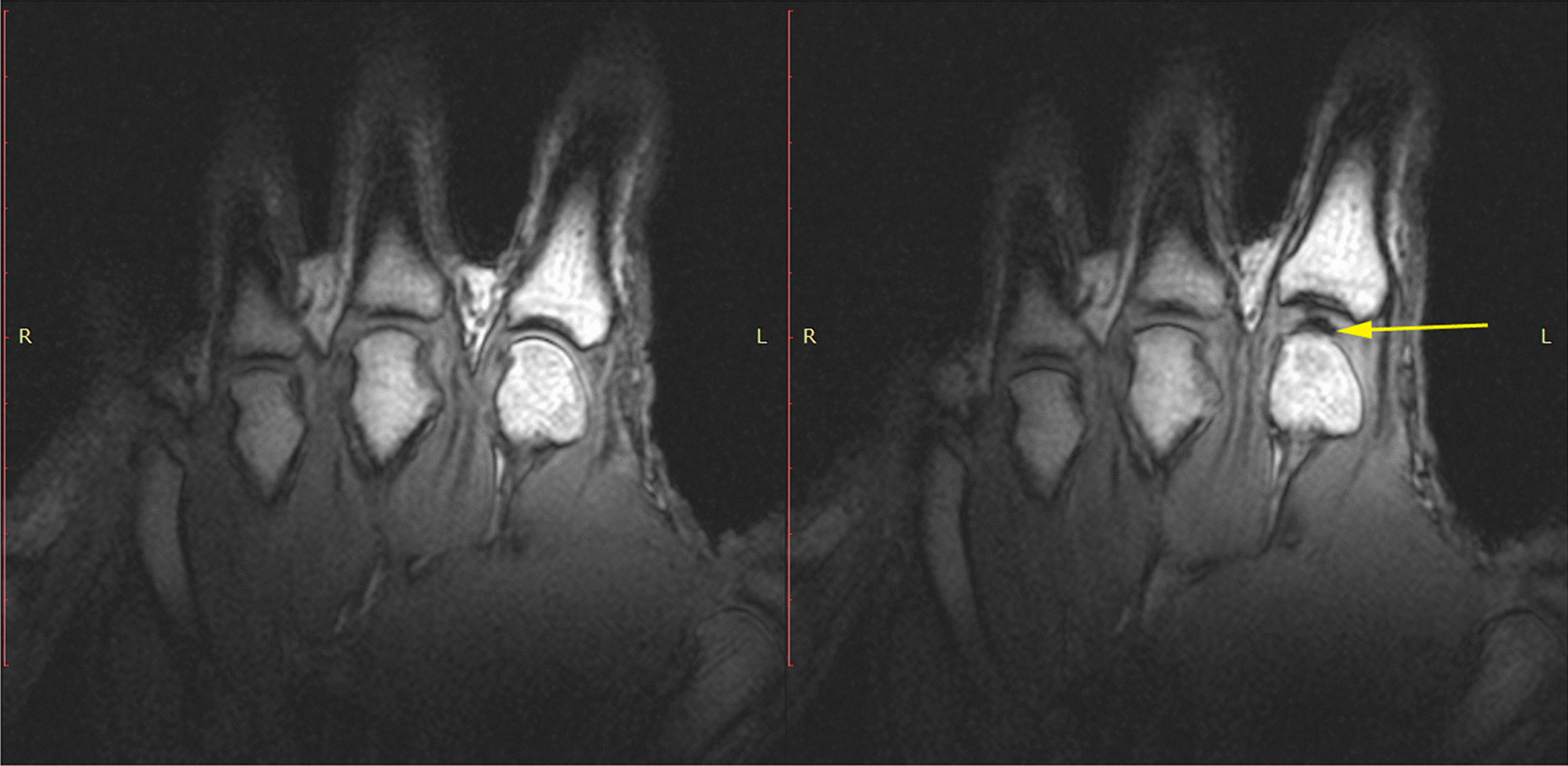
MR images before and after cavitation in an MCP joint. The original caption of this figure, reproduced from Herzog et al. [33], was “T1 static images of the left hand in the resting phase before cracking (left). The same hand following cracking with the addition of a post-cracking distraction force (right). Note the dark, intraarticular void (yellow arrow)”

In 2015, Kawchuk and colleagues in Canada [33] published beautiful images of cavitation bubble formation using high-frequency MR imaging (Fig. 11). Despite conclusions to the contrary, we know from the work of Watson and Mollan [29] some 25 years earlier (which showed that the bubble appears within 8.3 ms), that valid deductions relating to the precise timing of bubble formation or collapse cannot be drawn from these data, since they were acquiring their MR images every 310 ms (i.e., 3 frames per second). Nevertheless, Kawchuk et al. [33] provided further evidence that bubbles were formed within the synovial fluid of a joint as a result of its articular surfaces being separated. The real-time video that the group produced and published alongside their study report is particularly worthy of attention.

**Fig. 12:**
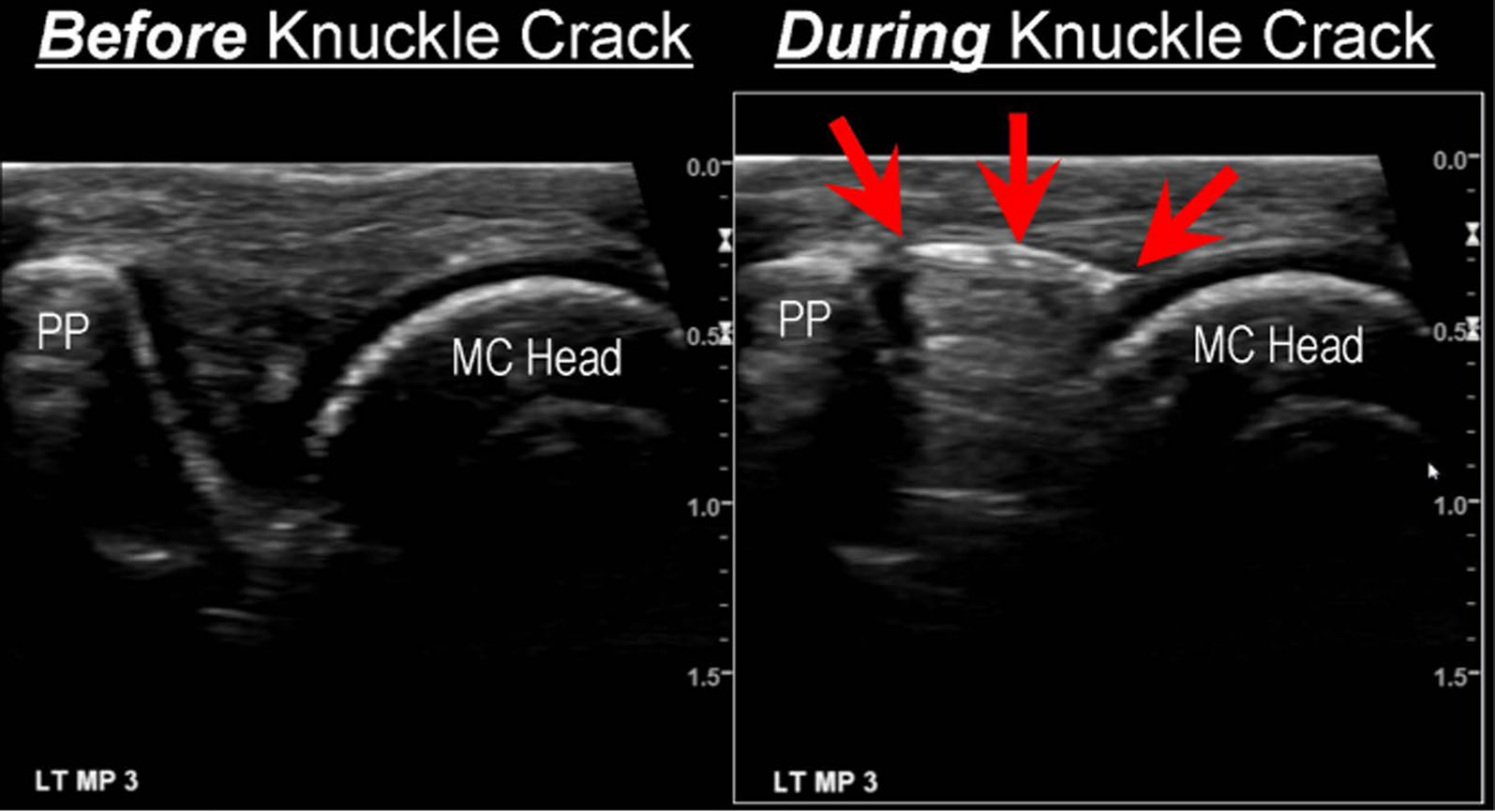
Ultrasound images before and after cavitation in an MCP joint. From Boutin et al. [34]

In 2016, another ultrasonography study was published [34] and this time the authors, Boutin and colleagues from the USA, *did* report on the timing of bubble formation. The sonograph images were acquired every 4.3 ms (232 frames per second, nearly twice the rate captured using x-ray by Watson and Mollan [29]), and bright ‘flashes’ were clearly visible within the joint space, occurring at some point between the first and second images (i.e., 4.3 and 8.6 ms) after the audible crack was recorded. Moreover, Boutin et al. [34] imaged some 400 different MCP joints from 40 asymptomatic adult subjects, making this the biggest study of the joint cracking phenomena to date. Again, the stationary image in Fig. 12 does not do justice to the amazing real-time videos made available by the authors alongside their published report.

All of the imaging studies discussed here have their methodological limitations. Repeating the high-frequency x-ray approach, used for the first time by Watson and Mollan [29] in the 1980’s, would certainly provide images at a very high frequency. However, radiographs are a poor imaging medium with which to make enquires about joint capsules and the dynamics of synovial fluid within, since they cannot capture much detail of either. Sonographs captured using ultrasound will record both fluid and capsules, as superbly demonstrated by Boutin and his team, and can do so at sub-millisecond frequency but bony structures preclude us from gaining a full cross-sectional profile of the joint. On the other hand, MR images show *all* of these structures well, and Kawchuck and colleagues [33] conceived an excellent methodology to separate joint surfaces within an MR environment, but we are still a long way from acquiring MR images at millisecond frequency. Undoubtedly, as imaging technology advances, our knowledge and understanding of both bubble formation and collapse, and capsular deformations during synovial fluid cavitation will continue to improve.

Despite the advances in knowledge over the past 15 years, there are some additional predictions made by the corrected model (Table 2) that have yet to be tested. For example, the corrected model predicts that joint distraction (and subsequent cavitation) should be achieved more easily when the joint configuration is in, or near to, its neutral configuration. Put another way, less force per unit separation (or kinetic energy) should be required to achieve cavitation when the joint is at, or close to, its neutral configuration. No study has yet looked at distraction forces required to achieve cavitation in a synovial joint when it is positioned in different angles from neutral. However, there is some empirical support for increased *articular surface separation* (the indisputable precursor to cavitation) being achieved when a glenohumeral joint is placed in a position of ‘maximal laxity’ compared to end range positions [35, 36]. Hence, this prediction of the corrected model has performed well so far.

## Summary

For manipulation, this paper has addressed arguably the most fundamental question that can be asked about any therapeutic intervention: *what is it*? In answering this question, the prevailing model of joint manipulation (of Sandoz) has been presented, alongside the research on the phenomenon of ‘joint cracking’ that led to its development. Research published since has also been covered in detail. Without exception, every single study on the subject shows that Sandoz’s model is fundamentally flawed. More concerning, the flaw in this model makes worrying predictions that could lead to dangerous clinical decisions. These predictions have been used (fairly) by observers to criticise the use of manipulation as a therapeutic intervention.

Despite its flaw, Sandoz’s model has been highly influential. Retaining it is dangerous though, both for patients and for the reputation of manipulation as a therapeutic intervention. Moreover, retaining it in the knowledge of its flaw is unethical and will damage trust in the professional groups that use manipulation. Accordingly, Sandoz’s model should be removed from clinical training curricula with immediate effect. Thankfully, this removal will not leave a void. A corrected model, first published more than 15 years ago [24], makes predictions in line with *all* available empirical data published before and since its conception. This corrected model also looks likely to fair well as new predictions are tested. It provides reassuring answers to critics, which should help with its acceptance by professional bodies and teaching institutions. Additionally, the corrected model should help clinicians to better judge the likely mechanisms of action, indications, and contraindications for the use of manipulation.

At this point, it is worth re-reading the definitions of manipulation listed in Table 1, which were presented at the outset of this paper. When doing so, it becomes obvious that these definitions have inherited the flaw from Sandoz’s erroneous model. A next logical step will be to address a long overdue call [1] for a better, empirically derived definition, which will be consistent with the corrected model and serve to improve both the teaching of manipulation and its implementation as a safe and useful tool in musculoskeletal health care.

## Conclusions

The prevailing model of joint manipulation (of Sandoz) is fundamentally flawed and potentially dangerous. It should be universally replaced, with immediate effect, by a corrected model, which was first published more than 15 years ago.

## Data Availability

Not applicable (review article)
