# Correction: Why is the prevailing model of joint manipulation (still) incorrect?

**DOI:** 10.1186/s12998-023-00476-2

**Published:** 2023-01-19

**Authors:** David W. Evans

**Affiliations:** 1 Centre of Precision Rehabilitation for Spinal Pain, School of Sport, Exercise and Rehabilitation Sciences, University of Birmingham, Edgbaston, Birmingham, B15 2TT UK; 2 Research Centre, University College of Osteopathy, London, UK

**Correction: Chiropractic & Manual Therapies (2022) 30:51** https://doi.org/10.1186/s12998-022-00460-2

Following the publication of the original article [1], two errors were identified in the article. The updated text is given below, and the change has been highlighted in bold typeface.

Under the **Cracking joints** header:

After this crack, with increasing load the line returns to a near horizontal gradient once again, until it reaches a maximum separation of approximately **4** mm; a relatively large separation for an MCP joint!

In the legend of **Fig. 11**:

MR images before and after cavitation in an MCP joint. The original caption of this figure, reproduced from **Kawchuk** et al. [33], was “T1 static images of the left hand in the resting phase before cracking (left). The same hand following cracking with the addition of a post-cracking distraction force (right). Note the dark, intraarticular void (yellow arrow)”.

The original article [1] has been corrected.

## References

[1] Evans DW (2022). Why is the prevailing model of joint manipulation (still) incorrect?. Chiropr Man Therap.

